# Enhancing Differential Diagnosis Related to Oxidative Stress, Nitrous Oxide, and Nutrition by Rapid Plasma Homocysteine Measurement

**DOI:** 10.3390/jox14040075

**Published:** 2024-09-27

**Authors:** Guillaume Grzych, Farid Zerimech, Benjamin Touze, Clarence Descamps, Marie-Adélaïde Bout, Marie Joncquel, Claire Douillard, Isabelle Kim, Céline Tard, Thierry Brousseau

**Affiliations:** 1 CHU Lille, Service Biochimie Automatisée-Protéines, F-59000 Lille, Franceisabelle.kim@chu-lille.fr (I.K.); thierry.brousseau@chu-lille.fr (T.B.); 2 CHU Lille, Service Hormonologie Métabolisme Nutrition Oncologie, F-59000 Lille, France; farid.zerimech@chu-lille.fr (F.Z.);; 3 CHU Lille, Service d’Endocrinologie et des Maladies Métaboliques, Reference Center for Inherited Metabolic, F-59000 Lille, France; 4 CHU Lille, Service Neurologie U1172, Centre de Référence des Maladies Neuromusculaires Nord/Est/Ile-de-Frace, F-59000 Lille, France

**Keywords:** homocysteine, mass spectrometry, immunoanalysis, nitrous oxide, nutrition, vitamin B12, neurology, oxidative stress, nutrition

## Abstract

Background: Historically used as a marker for inherited disorders, the current interest in plasma homocysteine measurement lies in its ability to provide valuable information about the metabolic and nutritional status of patients. Specifically, nitrous oxide (N_2_O) abuse can lead to functional vitamin B12 deficiency by oxidation and increase oxidative stress, resulting in elevated plasma homocysteine levels, which mimic neurological conditions such as Guillain–Barré syndrome. Rapid identification of hyperhomocysteinemia is crucial for timely intervention and avoiding costly, unnecessary treatments. Objective: This study evaluates the performance of a rapid immunoassay technique (Snibe) compared to mass spectrometry (LC-MS/MS) for measuring plasma homocysteine levels in patients with nitrous oxide abuse and non-inherited caused of elevated homocysteine, aiming to enhance differential diagnosis related to oxidative stress. Methods: 235 patients from Lille University Hospital were included. EDTA blood samples were collected and analyzed using both rapid immunoassay (Snibe) and LC-MS/MS. Neurological assessment was performed using the peripheral neuropathy disability (PND) score. Results: Firstly, significant elevations in plasma homocysteine levels were observed in patients abusing nitrous oxide measured by LC-MS/MS. Secondly, the immunoassay provided rapid results, essential for early clinical decision-making, but tended to underestimate high values compared to LC-MS/MS. A good correlation was found between the methods for low and moderate values. Conclusion: The immunoassay tended to underestimate high-value samples compared to LC-MS/MS, which is a common problem with the competitive methodology. The rapid immunoassay technique is effective for initial screening and early intervention, aiding in the differential diagnosis of conditions related to oxidative stress. Therefore, it is recommended to use the CLIA method for initial screening and confirm with mass spectrometry if there are abnormal samples. Integrating both techniques can enhance diagnostic accuracy and improve patient outcomes.

## 1. Introduction

Homocysteine is a sulfur-containing amino acid that is an intermediate in the metabolism of methionine. It is normally present in low concentrations in plasma, but its accumulation can indicate various metabolic and pathological disorders. Hyperhomocysteinemia, a condition characterized by elevated levels of homocysteine in the blood and relative to disturbance in antioxidant imbalance [1], has been associated with an increased risk of cardiovascular diseases, strokes [2], and neurodegenerative disorders. These associations have led to a growing interest in measuring plasma homocysteine levels in medical biology, both for its potential role as a diagnostic marker and for its implications in therapeutic monitoring.

Historically used for the diagnosis of inherited disorders (i.e., cystathionine beta synthase (CBS) deficiency), the current interest in plasma homocysteine measurement lies in its ability to provide valuable information about the metabolic and nutritional status of patients. Elevated homocysteine levels can indicate deficiencies in vitamins B6, B12, and folic acid [3,4], which are essential cofactors in its metabolism. Therefore, measuring homocysteine can be used to assess the effectiveness of nutritional interventions and to identify individuals at risk of vitamin deficiencies. Moreover, epidemiological studies have demonstrated that hyperhomocysteinemia is more often related to nutrition deficiency than inherited disorders and is an independent risk factor for cardiovascular diseases, highlighting the importance of this biomarker in the prevention and management of these pathologies.

Recent studies have highlighted the critical need for rapid plasma homocysteine measurement in cases of nitrous oxide abuse [5,6,7], especially for the differential diagnosis of Guillain–Barré syndrome. Nitrous oxide abuse is typically associated with recreational use due to its euphoric and dissociative effects. It is frequently consumed in social settings, such as parties or clubs, where it is inhaled directly from small cartridges or balloons. Its appeal lies in its accessibility, low cost, and rapid onset of effects. However, regular use can lead to neurological complications and metabolic disturbances, particularly through inactivation of vitamin B12. This results in increased homocysteine levels, which are associated with neurotoxicity and other health issues. Awareness and early detection are crucial for managing these risks effectively. Nitrous oxide abuse can lead to vitamin B12 deficiency [8], which subsequently causes hyper-elevated homocysteine levels [9]. The neurological symptoms resulting from this can mimic those of Guillain–Barré syndrome, including subacute numbness, tingling, ataxia, and weakness. However, the underlying mechanisms differ significantly, necessitating very distinct treatment approaches.

Early detection of elevated homocysteine levels can be pivotal in distinguishing between nitrous oxide-induced neurological damage and Guillain–Barré syndrome [10]. The team of Fortanier et al. demonstrated the critical importance of differentiating between nitrous oxide abuse and Guillain–Barré syndrome. However, they only used total sera vitamin B12 levels and not plasma homocysteine, because they considered that it was not possible to obtain urgent results for homocysteine related to mass spectrometry availability. Therefore, it is essential to develop rapid methods to improve diagnostic accuracy. Moreover, isolated measurement of total vitamin B12 could be complicated and related to the self-medication of patients consuming nitrous oxide.

In this context, rapid diagnosis and etiology of high homocysteine are crucial to prevent diagnostic uncertainty and unnecessary, expensive investigations, such as nerve conduction studies and cerebrospinal fluid analysis, which are typically employed to diagnose Guillain–Barré syndrome and even inappropriate treatments such as IgIV. Prompt identification of elevated homocysteine levels allows for timely intervention, such as vitamin B12 supplementation, potentially reversing the neurological symptoms associated with nitrous oxide abuse and initiating addiction follow-up of patients [11].

By avoiding diagnostic delays and the associated costs of extensive testing, rapid homocysteine measurement not only improves patient outcomes but also reduces the burden on healthcare systems. This approach underscores the importance of incorporating plasma homocysteine testing in the initial assessment of patients presenting with neurological symptoms and a history of nitrous oxide use.

The rapid measurement of plasma homocysteine levels is not only critical in cases of nitrous oxide abuse [10] and metabolic disorders but also in a broader range of clinical scenarios. Elevated homocysteine has been linked to an increased risk of cardiovascular diseases [12], including heart attacks and strokes [13], as well as neurodegenerative conditions such as Alzheimer’s disease [14]. In acute settings, such as emergency departments, timely identification of elevated homocysteine levels can be crucial for the immediate management and treatment of patients presenting with symptoms of acute cardiovascular events or unexplained neurological deficits. Rapid homocysteine testing can aid in the early diagnosis and differentiation of these conditions, guiding prompt therapeutic interventions and potentially improving patient outcomes. Moreover, in routine clinical practice, rapid results can facilitate the monitoring of patients on treatments aimed at lowering homocysteine levels, enabling timely adjustments to therapy and reducing the risk of long-term complications [15]. Thus, the implementation of rapid homocysteine testing methods extends beyond specific intoxications and metabolic disorders, playing a vital role in the comprehensive assessment and management of various acute and chronic conditions.

The aim of this study is, first, to show the interest in homocysteine measurement compared to solely vitamin B12 in the diagnosis of nitrous oxide abuse and second to evaluate a rapid immunoassay technique for measuring plasma homocysteine levels compared to the conventional mass spectrometry method, which, although highly accurate, is often too slow for timely decision-making in this pathological context. Mass spectrometry, while considered the gold standard for homocysteine measurement due to its precision and sensitivity, involves complex sample preparation and lengthy analysis times (days to weeks), rendering it impractical for urgent clinical scenarios. Conversely, immunoassay techniques offer the advantage of faster turnaround times, potentially providing critical diagnostic information within a clinically relevant timeframe (hours to days) [16].

This study will compare the performance of the rapid immunoassay technique to mass spectrometry in terms of accuracy, speed, and practicality in a clinical setting. By demonstrating the efficacy of the immunoassay method, we aim to establish a reliable and expedient diagnostic tool that can facilitate early intervention and improve patient outcomes in cases of nitrous oxide abuse, ultimately reducing the risk of diagnostic delays and minimizing unnecessary healthcare expenditures.

## 2. Materials and Methods

### 2.1. Patient Population

This study included patients from routine Lille Hospital University and from the BALON clinical trial study (NCT05540561) presented to the emergency department with suspected nitrous oxide abuse. EDTA blood samples were collected from each patient upon their arrival at the emergency service. For patients from the BALON study, neurological evaluation was performed using the peripheral neuropathy disability (PND) score to assess the severity of neurological symptoms [6]. The inclusion criteria for the study required that patients have a homocysteine prescription or a documented history of nitrous oxide use and present with neurological symptoms consistent with those observed in vitamin B12 deficiency or Guillain–Barré syndrome. We also included patients from our routine laboratory practice with homocysteine prescriptions and some patients with different causes of high homocysteine (nutritional deficiencies, CBS deficiencies, etc.) levels excluding nitrous oxide consumption to compare with non-nitrous oxide users, including patients with other causes of elevated homocysteine.

### 2.2. Sample Collection and Handling

Upon arrival at the emergency department, each patient underwent a standardized neurological assessment using the PND score. Blood samples were drawn into EDTA tubes to prevent coagulation and were immediately stored at 4 °C. The samples were then transported to the laboratory for plasma homocysteine analysis within two hours of collection to ensure sample integrity.

### 2.3. Vitamin B12 Measurement

In addition to plasma homocysteine levels, serum vitamin B12 levels were measured for all patients. Blood samples were collected in serum-separating tubes and analyzed using an electrochemiluminescence immunoassay (ECLIA) on a Cobas analyzer (Roche, France). The reference range for vitamin B12 was considered to be more than 200 pg/mL.

### 2.4. Homocysteine Measurement Techniques

#### 2.4.1. Mass Spectrometry

For comparison, plasma homocysteine levels were first measured using liquid chromatography-tandem mass spectrometry (LC-MS/MS) as the gold standard method. Plasma samples were first prepared by protein precipitation. The prepared samples were then injected into the LC-MS/MS system. The separation was achieved using a reverse-phase column, and the detection was carried out in multiple reaction monitoring (MRM) mode. The entire process, including sample preparation and analysis on LC-MS/MS, took approximately one half-day to one day and needed both technician and analyzer available, and could take several days to weeks to be performed depending on the laboratory.

#### 2.4.2. Immunoassay Technique

The plasma homocysteine levels were next measured using a rapid immunoassay technique (MAGLUMI, Snibe, Shenzhen, China). This method involved the use of a commercially available homocysteine immunoassay kit, which employs antibodies specific to homocysteine. The immunoassay was performed according to the manufacturer’s instructions. Briefly, plasma samples were incubated with the antibody reagent, and the resulting antigen-antibody. The total assay time, from sample preparation to result, was approximately one hour and 30 min.

### 2.5. Data Analysis

The homocysteine levels obtained from both the immunoassay and LC-MS/MS were compared to assess the performance of the rapid immunoassay. Statistical analysis included calculating the mean, median, and standard deviation of homocysteine levels, as well as assessing the correlation between the two methods using the Pearson correlation coefficient. Additionally, Bland–Altman plots were used to evaluate the agreement between the immunoassay and LC-MS/MS results. Sensitivity, specificity, and predictive values of the immunoassay were also determined, with LC-MS/MS results serving as the reference standard. Values are expressed as mean ± standard deviation. Values of *p* < 0.05 were considered statically significant. Statistical differences were determined by the Student *t*-test or Mann–Whitney U test for two-group comparisons.

## 3. Results

### 3.1. Characterization of the Population

The study included a total of 235 patients. The demographic and clinical characteristics of the patients are summarized in Table 1. The mean age of the patients was 45.6 years. The gender distribution was 54% male and 46% female (Table 1).

We observed 63 samples with high plasma homocysteine (>50 µmol/L) in the group of patients without nitrous oxide consumption and corresponding to 42 vitamin deficiencies (B6, B9, or B12 deficiencies), 8 inherited diseases (CBS deficiencies), 7 methylenetetrahydrofolate reductase (MTHFR) polymorphisms, and 6 kidney diseases.

### 3.2. Isolated Serum Vitamin B12 Levels Is Not a Good Marker for Nitrous Oxide Abuse

**Figure 1:**
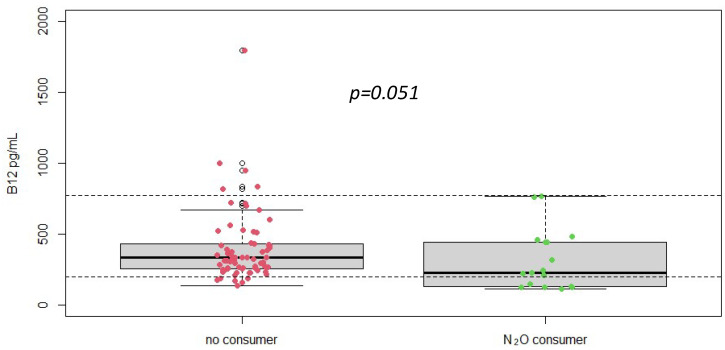
Plasma vitamin B12 concentrations according to different groups (No N_2_O consumer, *n* = 216 vs. N_2_O consumers, *n* = 19), *p*-value was obtained using Student *t*-test. Dotted line = reference range [197–771] pg/mL.

Serum vitamin B12 levels were measured in all patients. The mean vitamin B12 level was 477 ± 712 pg/mL, with a range from 109/5640 pg/mL (Figure 1). Despite the documented history of nitrous oxide abuse and elevated homocysteine levels, many patients had vitamin B12 levels within the normal reference range. This finding indicates that vitamin B12 measurement alone may not be sufficient for diagnosing nitrous oxide-induced neurological damage, as it does not consistently reflect the functional deficiency or the oxidative stress implicated in these cases.

### 3.3. Significant Increase in Plasma Homocysteine Levels in Cases of Nitrous Oxide Abuse

**Figure 2:**
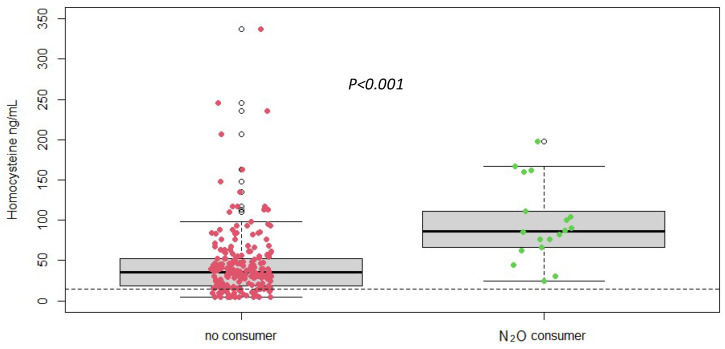
Plasma homocysteine concentrations according to different groups (No N_2_O consumer, *n* = 216 vs. N_2_O consumers, *n* = 19) (measured by LC-MS/MS), *p*-value was obtained using Student *t*-test. Dotted line = reference range < 14 ng/mL.

Plasma homocysteine levels were significantly (measured by LC-MS/MS) elevated in patients with nitrous oxide abuse compared to the normal reference range (Figure 2). The mean plasma homocysteine level in the study population was 47.88 ± 43.96 µmol/L, with values ranging from 5/337 µmol/L. This elevation underscores the impact of nitrous oxide on vitamin B12 metabolism and confirms the potential of homocysteine as a biomarker for detecting and monitoring the effects of nitrous oxide abuse and its importance for differential diagnosis. Next, the homocysteine assay needs to be optimized to provide rapid results for rapid diagnosis. We therefore want to evaluate a rapid method for measuring homocysteine in comparison with mass spectrometry.

### 3.4. Comparison between Mass Spectrometry and Immunoassay Could Diverge in High Homocysteine Values

**Figure 3:**
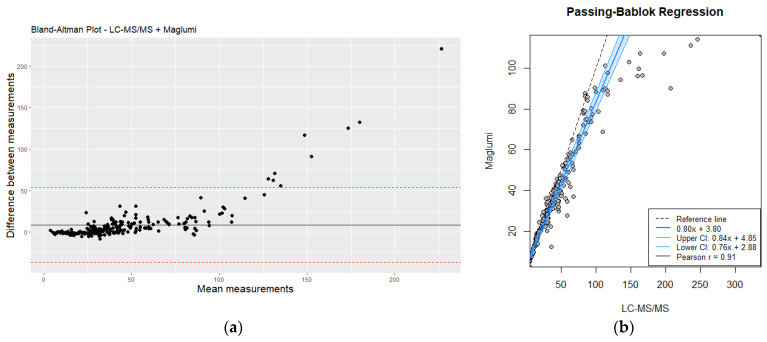
(**a**) Bland–Altman plot analysis of LC-MS/MS vs. Maglumi: evaluating homocysteine measurement differences; (**b**) comparative evaluation of LC-MS/MS and Maglumi homocysteine measurements using Passing–Bablok regression. Red line correspond to mean differences.

The comparison between plasma homocysteine levels measured by mass spectrometry (LC-MS/MS) and the rapid immunoassay technique revealed some discrepancies, particularly at higher concentration ranges. Figure 3 shows the distribution of homocysteine levels measured by both methods. While the immunoassay provided results within a clinically acceptable range for low to moderate homocysteine levels, it tended to underestimate the values at the higher end of the spectrum compared to LC-MS/MS.

### 3.5. Good Correlation in Low and Moderate Values until 30 µmol/L for Screening and Differential Diagnosis

**Figure 4:**
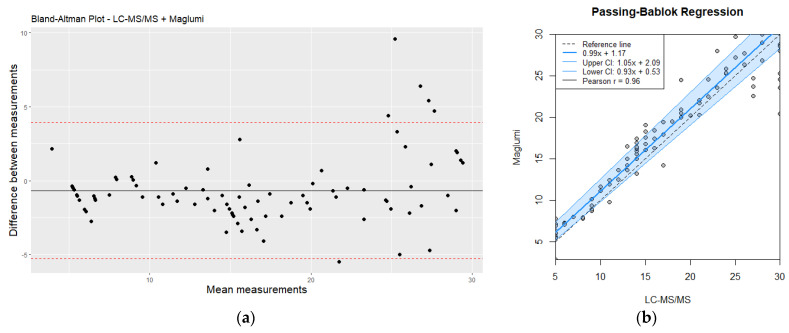
(**a**) Bland–Altman plot analysis of LC-MS/MS vs. Maglumi: evaluating homocysteine measurement differences (values < 30 µmol/L); (**b**) comparative evaluation of LC-MS/MS and Maglumi homocysteine measurements (values < 30 µmol/L) using Passing–Bablok regression. Red line correspond to mean differences.

Despite the observed discrepancies, there was a good overall correlation between the two methods for moderate and high homocysteine values, (r = 0.91, *p* < 0.05). Bland–Altman analysis (Figure 4) further illustrated the agreement and the limits of agreement between the two methods. The immunoassay demonstrated acceptable performance for rapid screening and initial assessment; however, LC-MS/MS remains the preferred method for precise quantification, particularly for higher homocysteine levels (>30 µmol/L) where accurate measurement is critical. This is especially important for clinical decision-making and for monitoring the effectiveness of therapeutic interventions.

### 3.6. Performances of Homocysteine Immunoassay Method

We assessed intra-assay precision by pooling plasma samples to create three pools at different concentration levels: approximately 10 µmol/L (low), 30 µmol/L (medium), and 60 µmol/L (high). Each pool was measured with 10 replicates per level in a single run. The concentrations of these levels were determined using LC-MS/MS. Intra-assay precision ranged from 0.73% to 2.21% CV (Table 2). Similarly, we assessed inter-assay precision by measuring plasma QC samples across 14 runs. Inter-assay precision was 2.75% and 3.73% for the low and medium levels of QC, respectively.

#### Dynamic Range

We assessed linearity by diluting a pool of four plasmas with high homocysteine concentrations, measured by LC-MS/MS, the reference method. Successive dilutions were performed from neat to 1:200, as shown in Table 3. The manufacturer’s designated lower limit for a reproducible signal was 0.8 µmol/L, and the upper limit was 90 µmol/L.

## 4. Discussion

This study aimed to evaluate the efficacy of a rapid immunoassay technique for measuring plasma homocysteine levels in patients with nutritional deficiencies or suspected nitrous oxide abuse, compared to the conventional mass spectrometry method. Our results demonstrated a significant increase in plasma homocysteine levels among the study population, highlighting the impact of nitrous oxide on vitamin B12 metabolism and the subsequent elevation of homocysteine. The rapid immunoassay provided timely results that are crucial for early intervention, although it showed some limitations at higher homocysteine concentrations.

The rapid immunoassay technique proved to be a valuable tool for the initial screening of elevated homocysteine levels in a clinical setting, as demonstrated by the excellent correlation with the gold standard measure technique. In specific cases of nitrous oxide abuse, timely identification of hyperhomocysteinemia is critical for distinguishing between nitrous oxide-induced neurological damage and other conditions such as Guillain–Barré syndrome [10]. Early detection allows for prompt vitamin B12 supplementation, potentially reversing the neurological symptoms [17] and preventing further complications such as thrombosis [7,11]. Moreover, the rapid turnaround time of the immunoassay facilitates immediate clinical decision-making, reducing diagnostic uncertainty and minimizing unnecessary, costly diagnostic procedures [18].

Our study also assessed serum vitamin B12 levels to determine their diagnostic value alongside homocysteine measurement. Interestingly, despite the presence of neurological symptoms and elevated homocysteine levels, many patients exhibited vitamin B12 levels within the normal reference range. This discrepancy suggests that vitamin B12 measurement alone may not provide a reliable indication of the oxidative stress and metabolic disruption caused by nitrous oxide abuse [19] and these data reinforce our previously published study which showed that homocysteine was a marker of consumption but not of clinical severity (where methylmalonic acid had shown a correlation with the clinical severity of patients) [6]. The functional deficiency of vitamin B12, which leads to elevated homocysteine, is more accurately captured by measuring plasma homocysteine levels directly. Thus, relying solely on serum vitamin B12 levels could result in missed or delayed diagnoses, underscoring the importance of homocysteine as a more sensitive and specific biomarker in this context [20].

The necessity for rapid plasma homocysteine measurement extends beyond cases of nitrous oxide abuse [21] and metabolic disorders to a wider range of clinical scenarios [22,23,24,25,26,27,28]. Elevated homocysteine levels are associated with an increased risk of cardiovascular diseases, including myocardial infarction and stroke [2]. The rapid immunoassay method evaluated and validated in this study will enable the detection of high homocysteine levels in these specific contexts, improving patient management significantly. In acute care settings, such as emergency departments, timely identification of hyperhomocysteinemia is crucial for the immediate management of patients presenting with symptoms of acute cardiovascular events or unexplained neurological deficits. Rapid homocysteine testing can facilitate early diagnosis and differentiation of these conditions, guiding prompt therapeutic interventions and potentially improving patient outcomes. This approach reduces diagnostic uncertainty, shortens hospital stays, and lowers healthcare costs, while enabling quicker and more effective treatment. Therefore, the implementation of rapid homocysteine testing methods, such as the immunoassay evaluated in this study, is essential not only for specific intoxications and metabolic disorders but also for enhancing the overall assessment and management of various acute and chronic conditions. This broader applicability underscores the clinical value of developing and utilizing rapid homocysteine measurement techniques in diverse medical contexts.

While the rapid immunoassay demonstrated a good overall correlation with mass spectrometry for low and moderate homocysteine levels, it tended to underestimate values at the higher end of the spectrum (>30 µmol/L). This discrepancy can be attributed to the inherent limitations of immunoassay techniques, such as potential cross-reactivity and reduced sensitivity at extreme concentrations. However, a value of 30 µmol/L could be an acceptable value because it concerns the majority of routine homocysteine in the laboratory. In contrast, mass spectrometry, with its high precision and sensitivity, remains the gold standard for accurate quantification of homocysteine levels, particularly in cases requiring precise measurement for clinical management.

The findings of this study underscore the importance of incorporating rapid homocysteine measurement in the initial assessment of patients presenting with clinical signs related to high homocysteine such as potential nutrition deficiency, thrombosis neurological symptoms, or a history of nitrous oxide use. The immunoassay technique offers a practical and efficient solution for initial screening, enabling healthcare providers to quickly identify cases of hyperhomocysteinemia and initiate appropriate interventions [29,30,31]. However, for cases with significantly elevated homocysteine levels, confirmatory testing with mass spectrometry and additional exploration such as methylmalonic acid and methionine is recommended to ensure accurate diagnosis and optimal patient care [32,33,34,35].

One limitation of this study is the relatively small sample size, which may affect the generalizability of the findings. Moreover, vitamin B12 deficiency can affect individuals of all ages and is present in individuals following a vegetarian/vegan diet [36,37]. Furthermore, advancements in immunoassay technology could potentially enhance the accuracy and reliability of rapid testing methods, reducing the need for confirmatory mass spectrometry in high-value cases.

## 5. Conclusions

In conclusion, this study highlights the critical role of rapid plasma homocysteine measurement in the clinical management of patients with nutrition disorders and nitrous oxide abuse. The rapid immunoassay technique provides a valuable tool for early detection and intervention, although mass spectrometry remains essential for precise quantification in high-value cases. Integrating these complementary methods into clinical practice can improve diagnostic accuracy, facilitate timely treatment, and ultimately enhance patient outcomes.

## Figures and Tables

**Table 1:** Clinical and biological characteristics of selected patients.

	All Patients	No-N_2_O Consumer	N_2_O Consumer
*n*	235	216	19
Age	45.6 ± 20	47.45 ± 19.73	24.58 ± 6.91
Sex (women/men)	108/127	101/115	7/12
Homocysteine levels	47.88 ± 43.96 µmol/L	43.85 ± 41.35 µmol/L	93.68 ± 47.86 µmol/L
Homocysteine min/max	5/337 µmol/L	24/167 µmol/L	5/337 µmol/L
Vitamin B12 Levels	477 ± 712 pg/mL	515 ± 780 pg/mL	312 ± 213 pg/mL
Vitamin B12 min/max	109/5640 pg/mL	136/5640 pg/mL	109/768 pg/mL

**Table 2:** Intra- and inter-assay precision of immunoassay in plasma and QC samples.

	Intra-Assay			Inter-Assay	
Levels	Low Sample	Medium Sample	High Sample	Low QC	Medium QC
n	10	10	10	14	14
Mean (pg/L)	10.6	31.2	60.4	4.2	14.2
SD	0.23	0.44	0.44	0.11	0.53
%CV	2.21%	1.39%	0.73%	2.75%	3.73%

SD: standard deviation, CV: coefficient of variation.

**Table 3:** Linearity of immunoassay method by successive dilution.

Plasmas Pooled (*n* = 4)	HCY (µmol/L) Expected	HCY (µmol/L) Obtained	Recovery
Pur	120	90.0	75%
1:2	60	56.0	93%
1:3	40	39.6	99%
1:4	30	30.0	100%
1:5	24	24.6	103%
1:8	15	13.3	89%
1:16	7.5	6.6	88%
1:32	3.75	4.4	117%
1:64	1.88	2.9	156%
1:100	1.2	2.5	207%
1:200	0.6	1.6	263%

## Data Availability

The data presented in this study are available in the article.
